# A survey on colonoscopy shows poor understanding of its protective value and widespread misconceptions across Europe

**DOI:** 10.1371/journal.pone.0233490

**Published:** 2020-05-21

**Authors:** Bharat Amlani, Franco Radaelli, Pradeep Bhandari

**Affiliations:** 1 Norgine Ltd., Medical Affairs, Harefield, United Kingdom; 2 Endoscopy Unit, Department of Gastroenterology, Valduce Hospital, Como, Italy; 3 Department of Gastroenterology, Portsmouth University Hospital, Portsmouth, United Kingdom; Rudjer Boskovic Institute, CROATIA

## Abstract

**Background:**

Colonoscopy is a valuable screening tool for colorectal cancer. However, patients experience anxiety when faced with attending a first colonoscopy, and negative attitudes may contribute to non-attendance. Few studies in Europe have explored these attitudes, despite increasing colorectal cancer incidence.

**Study aim:**

We conducted an online survey of the public in five European Union countries (France, Germany, Italy, Spain, and the UK), with the aim of understanding public knowledge of, perceptions of, and attitudes towards, colonoscopy and bowel preparation, amongst colonoscopy-naïve respondents. Attitudes towards colonoscopy were also gathered from colonoscopy-experienced patients.

**Methods:**

Survey answers were gathered from 2,500 colonoscopy-naïve respondents and 500 colonoscopy-experienced patients, divided equally between countries.

**Results:**

Across Europe, 72% of colonoscopy-naïve respondents showed receptiveness to colonoscopy if advised by their doctor to receive one, but only 45% understood its use to prevent colorectal cancer. Forty-three percent of colonoscopy-experienced respondents would still be embarrassed about having another colonoscopy, although 59% said that the experience had been better than expected. Colonoscopy-experienced respondents had greater aversion to bowel preparation than colonoscopy-naïve respondents (47% vs 26%), and 67% of colonoscopy-naïve respondents thought that only 1 litre of bowel preparation or less is required. Italians and the Spanish wanted more information than on average in Europe, while Germans had more realistic expectations of bowel preparation.

**Discussion:**

There are perceptual gaps amongst the public around the purpose of colonoscopies, the subjective experience of the colonoscopy procedure, and the quantity of bowel preparation needed. These concerns could be mitigated by better education and using lower-volume bowel preparation techniques.

**Conclusion:**

Europeans would have a colonoscopy, but its preventive medical purpose is poorly understood and there are misconceptions around the process. Further education about the procedure, its benefits and bowel preparation is vital to improve understanding and compliance.

## Introduction

Colorectal cancer (CRC) is the third most common malignancy worldwide, and the second leading cause of cancer-related death [1]. CRC rates are also increasing, believed to be due to the advance of a western lifestyle; CRC is found at higher rates in more developed countries. In Europe for instance, in countries such as Italy, Spain and the UK, the incidence of CRC has increased over a recent 10-year period. In contrast with Europe, the incidence of CRC has decreased over 10 years in the USA, though the causes of this are ill-defined [2].

Colonoscopy is the gold-standard method of screening for CRC, and it has been shown to reduce mortality from CRC in the general population by 22–31% [3]. However, attendance rates for colonoscopy are poor; in the European Union, compliance with colonoscopy referral ranges between 64% and 92% [4]. Numerous barriers to compliance have been investigated in the literature, and summarised in prior systemaic reviews [5,6]. Different societal levels can be highlighted, as points at which such barriers occur: policy-level (e.g. limitations of how colonoscopies are organised), provider-level (e.g. limitations of what instruction is given to patients), demographic-level (e.g. obstacles arising from socioeconomic status) [7], and patient-level (e.g. health status and attitude) [5]. Health literacy, for example, may be influenced by both information provision and patient attitude to learning.

Given the increasing incidence rate of CRC in Europe, we take a special interest in the personal attitudes towards colonoscopy itself, as interviews that we conducted with patient advocacy groups, healthcare professional groups and academics in Europe highlighted public misconceptions about, and fear of, the colonoscopy process. Previous European studies have shown that embarassment (Spain) [6] and fear of pain (France) [8] are barriers to compliance with colonoscopy attendance. Anxiety is natural when anticipating an invasive procedure such as a colonoscopy, and 20–30% of patients attending colonoscopies in the US have also reported anxiety in the clinically significant range [9,10].

Determining the specific anxieties around colonoscopy in Europe, and their validity, could provide insights into what particular education is needed to improve compliance, reducing the financial and resourcing losses for healthcare systems due to non-attendance, and potentially providing a long-term beneficial impact for patients. A single negative colonoscopy screening result is associated with a 46% lower risk of CRC for patients, and an 88% lower risk of related deaths at the current guideline-recommended 10-year rescreening interval, likely due to the very slow development of CRC from its precancerous state [11,12].

Previous surveys of attitudes to colonoscopy in Europe included a survey of 1,458 colonoscopy-naïve 50–69 year olds registered with general practices in the UK [13], and a survey of 505 women at an Italian cancer prevention centre [14]. The UK study found that 23% of responders find the idea of screening colonoscopy either ‘unacceptable’ or ‘very unacceptable’, but patients were significantly less likely to have a negative attitude of colonoscopy if symptomatic [13]. The Italian study found that women thought colonoscopy was more embarrassing than a Pap test, and fear, embarrassment, anxiety, fear of results, and annoyance around preparation was more common for colonoscopy than for mammograms; perception of pain or embarassment led to 70% lower odds of compliance, amongst those women referred for a colonoscopy [14]. Meanwhile, US and Canadian outpatients studies found that patients were also concerned about pain and embarrassment, as well as the results and various aspects of the bowel preparation [9,10,15,16]. However, all of these studies reflect colonoscopy-experienced or volunteer patients, who may be more receptive to, or knowledgeable of, healthcare intervention than the population at large.

In the present study, we performed a market research survey into public attitudes towards colonoscopy in five European countries. This presents, to our knowledge, both the first pan-European survey and the first public attitudes survey to assess: specific beliefs about the colonoscopy process, attitudes towards patient education, specific anxieties around colonoscopy, and factual knowledge about the procedure. Data were gathered from colonoscopy-naïve and -experienced respondents in order to validate expectations.

## Materials and methods

### Study design

This survey study was conducted by the market research agency GfK (now Ipsos Healthcare), based in Germany. The methodology complied with German Market Research Guidelines, European Society for Opinion and Market Research e.V. (ESOMAR) guidelines, the Working Group of German Market and Social Research (ADM) guidelines, and European Pharmaceutical Market Research Association (EphMRA) guidelines. As per EphMRA guidelines 3.5 and 3.6, this study complied with the European Federation of Pharmaceutical Industries and Associations (EFPIA) requirements for market research (as opposed to clinical research), and thus did not require clinical research ethics committee approval. The study complied with all German data protection regulations, and investigators were trained in adverse event reporting by the British Healthcare Business Intelligence Association (BHBIA).

From December 2017 to January 2018, the agency conducted an online survey in five European Union countries (EU5)–France, Germany, Italy, Spain, and the UK–with the aim of identifying and understanding public attitudes towards colonoscopy. The survey was anonymous, and was sent to members of the public who had volunteered to take part in surveys in exchange for points towards consumer gift vouchers.

First, a preliminary survey of five questions about respondent characteristics was sent to adults aged 18–70 years, and targeted quotas of 500 adults in each country who had never had a colonoscopy (2,500 colonoscopy-naïve respondents) and 100 adults in each country who had had a colonoscopy in the last five years (500 colonoscopy-experienced respondents). Quotas were filled such that their sample compositions matched the national demographic profiles in each country in terms of age, gender, administrative region, and occupational status. For example, each country was split into administrative regions (France: 13; Germany: 16; Italy: 21; Spain: 18; UK: 12) and quotas were matched to represent the proportional population of each region. Quota sizes were chosen as a compromise between statistical robustness and logistical feasibility; for example, a survey result of 80% for 500 respondents has a confidence interval range of 7% at p = 0.05, but a doubling of the quota size, for example, only reduces the confidence interval range by a relative 29% (to 5%).

Currently, there is no standardized questionnaire to evaluate the burden of colonoscopy or bowel preparation, so the agency developed questions for the main survey based on the feedback of the initial interviews conducted with patient advocacy groups, healthcare professional groups, and academics around misconceptions in colonoscopy. Colonoscopy-naïve respondents were given 24 questions to answer, covering respondents’ personal attitudes towards colonoscopy, factual knowledge, and perceptions of colonoscopy, and preferences for obtaining information about colonoscopy. Colonoscopy-experienced respondents were given 32 questions covering respondents’ perceptions of their previous colonoscopy, and personal attitudes towards another colonoscopy.

For some questions, respondents were provided with answers to choose between, for example the question ‘Which of the following do you think colonoscopy is used for?’ had answer choices that included ‘Preventing bowel cancer’ and ‘Ongoing monitoring of people who are known to have a bowel problem’. For other questions, respondents were asked to indicate their level of agreement with a statement about colonoscopy between ‘strongly disagree’ and ‘strongly agree’, on a 1 to 5 scale, for example ‘Having a colonoscopy is painful’ and ‘Even if I was worried or embarrassed, I would still have a colonoscopy’. For a full list of questions and answer choices, see the S1 Appendix.

### Statistics

Responses were summarized using descriptive statistics. Odds ratios were calculated for the odds of agreement with a statement after having had a colonoscopy vs not having had a colonoscopy. Endorsments and agreements were compared between each country and the EU5 average, using a 2-sample t-test with a 95% confidence level. Figures of results were created in Adobe® Illustrator.

## Results

A total of 53,795 people were invited to take the preliminary survey; 18,650 people followed the link to complete the survey and subsequently the target quota of 3,000 respondents was filled. Of the other 15,650 people who followed the link to the preliminary survey but who were not included in the final quota: 285 people were excluded as they had had a colonoscopy over five years ago; 14,325 people were not given access to the main survey as the quota for their demographic was already filled at the time of their preliminary response; and 1,040 people did not complete their response before their quotas were filled.

Results are broken down into: (i) respondent characteristics; (ii) knowledge about colonoscopies (colonoscopy-naïve respondents’ knowledge, or expectations compared with the colonoscopy-experienced respondents’ experience); (iii) attitudes to the colonoscopy process (all respondents’ perceptions of hypothetical scenarios); and (iv) opinion of information provided before colonoscopy.

### (i) Respondent characteristics

Characteristics of the respondents are shown in Table 1.

**Table 1:** Respondents’ characteristics (information in brackets signifies: Country with the lowest value; country with the highest value).

**Characteristic**	**Colonoscopy-naïve (n = 2,500)**	**Colonoscopy-experienced (n = 500)**
**Female**	55%	43%
	(54%, UK; 58%, Germany)	(40%, France; 45%, Spain)
**Age (mean ±s.d. years)**	42.3 ±14.0	47.0 ±14.6
	(39.8 ±12.6, Germany; 43.7 ±14.4, UK)	(42.0 ±14.3, UK; 50.6 ±13.8, France)
**Aged ≥41 years**	54%	65%
	(49%, Germany; 57%, Italy)	(47%, UK; 74%, France)
**In full- or part-time employment**	58%	65%
	(51%, Italy; 71%, UK)	(56%, France; 79%, UK)
**Retired**	9%	17%
	(5%, Germany; 13%, Spain)	(9%, UK; 29%, France)
**Time since prior colonoscopy**	*N/A*	0–1 year ago: 27%;
		1–2 years ago: 43%;
		2–3 years ago: 21%;
		3–5 years ago: 9%
**Reason for prior colonoscopy**	*N/A*	Routine screening: 30%;
		Symptomatic: 52%;
		Ongoing health condition: 10%;
		Other/Unknown: 7%

s.d. = standard deviation.

### (ii) Knowledge about colonoscopies

#### Knowledge about colonoscopies: Purpose

Sixty-seven percent of colonoscopy-naïve respondents stated that they knew someone who had had a colonoscopy. Eighty-seven percent stated that they either had ‘detailed knowledge’ (13%), a ‘good idea’ (34%), or knew ‘a little’ (40%) about colonoscopy; only 13% stated that they did not know what a colonoscopy is.

Colonoscopy-naïve respondents had various views on why colonoscopies are conducted, with the most common perception (78%) being that colonoscopies are used to diagnose bowel diseases. Sixy-five percent of respondents thought colonoscopies are used for bowel cancer screening, and 48% thought that they are used for the ongoing monitoring of people who are known to have a bowel problem. Only 45% of respondents thought they are used for the prevention of bowel cancer, and even fewer, 35%, for the removal of an unusual bowel growth. The only inter-country deviations of more than 10% from the EU5 averages were UK and French knowledge of colonscopy’s prevention of bowel cancer, at 26% and 56%, respectively.

#### Knowledge about colonoscopies: Sedation, duration, and pain

Thirty percent of colonoscopy-naïve respondents agreed that ‘you need to be unconscious to have a colonoscopy’ (lowest: 16%, UK; highest: 42%, Spain), while 39% disagreed (lowest: 20%, Spain; highest: 57%, UK). Thirty-four percent of the colonoscopy-experienced respondents reported having had full sedation during the procedure (lowest: 12%, UK; highest: 70%, France). Additionally, 11% of colonoscopy-naïve respondents believed that a colonoscopy lasted <15 minutes, 38% for 15–30 minutes, 24% for 30–60 minutes, 5% for more than one hour, 1% for more than two hours, 1% for more than three hours, and 21% did not know.

Forty percent of colonoscopy-naïve respondents thought that having a colonoscopy is painful (lowest: 32%, Germany; highest: 53%, Italy), whereas 25% of the colonoscopy-experienced respondents thought the same (lowest: 9%, Germany; highest: 39%, Italy); odds ratio 0.50. Further, 59% of the colonoscopy-experienced respondents thought that the colonoscopy experience was better than expected (lowest: 48%, France; highest: 71%, Spain) and 35% thought it was as expected (lowest: 22%, Spain; highest: 46%, France).

#### Knowledge about colonoscopies: Bowel preparation

**Fig 1:**
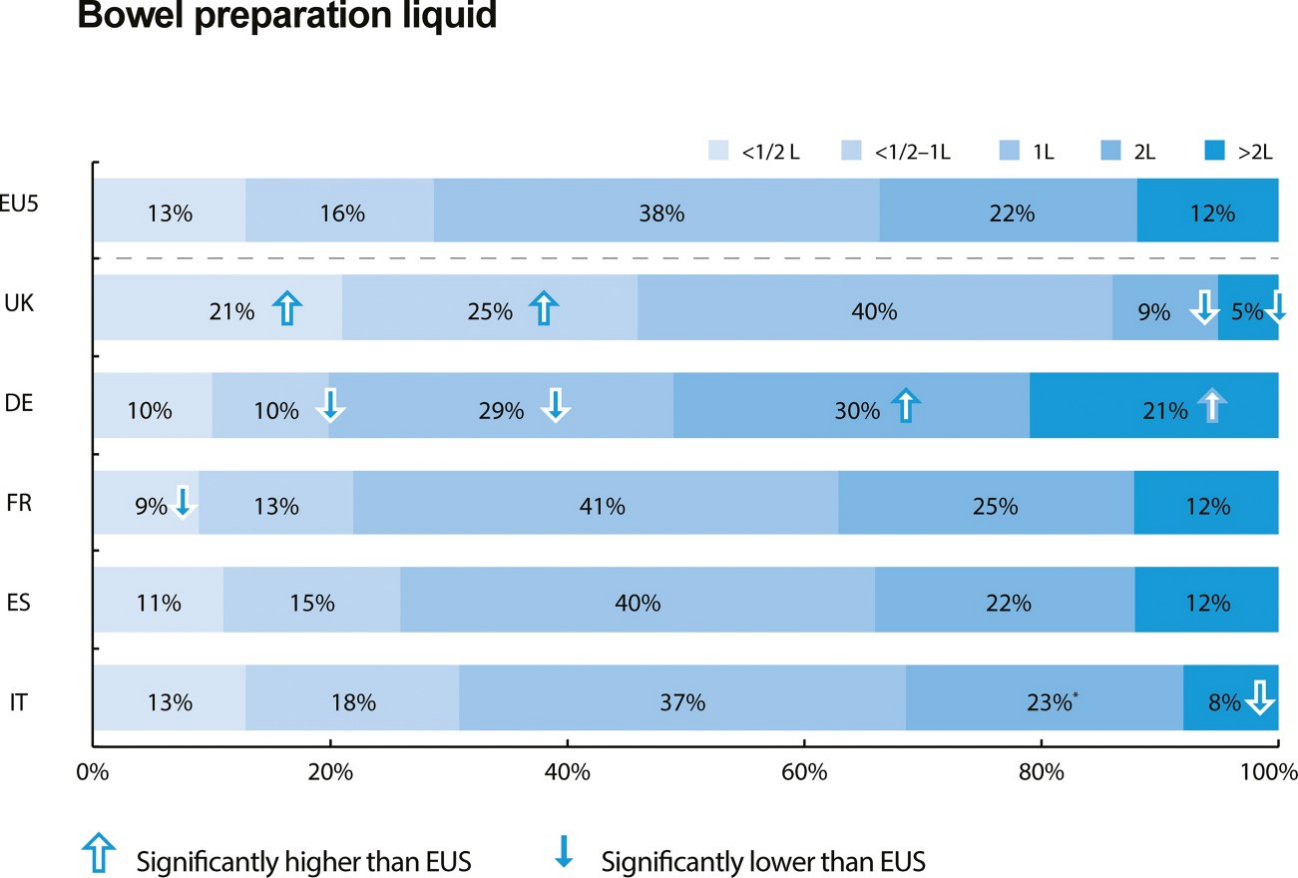
The amount of bowel preparation liquid that colonoscopy-naïve respondents think a patient needs to drink prior to the procedure (by country). Significance denotes p<0.05. EU5 = five European Union countries, UK = United Kingdom, DE = Germany, FR = France, ES = Spain, IT = Italy.

Sixty-seven percent of colonoscopy-naïve respondents believed that 1 litre of bowel preparation or less is required (choices: <0.5L, 0.5–1L, 1L, 2L, or >2L), although there were significant variations in belief between countries (Fig 1). For example, UK respondents had the lowest expectation for bowel preparation volume required (86% believed that 1 litre or less is required), while German respondents showed the highest expectation (51% believed that 2 litres or more is required).

### (iii) Attitudes to the colonoscopy process

#### Attitudes towards having a colonoscopy

Attitudes towards having a colonoscopy for colonoscopy-naïve and colonoscopy-experienced respondents, and odds ratios for attitudes of respondents with colonoscopy experience, are shown in Table 2. The majority of colonoscopy-naïve respondents would be receptive to a colonoscopy if it found a problem early (82%) or if their doctor recommended it (72%), and would attend even if they were worried or embarrassed (67%).

**Table 2:** Respondents’ attitudes towards having a colonoscopy (information in brackets signifies: The range of responses across the five countries surveyed; country with the lowest agreement with statement; country with the highest agreement with statement).

**Agreement with statement**	**Colonoscopy-naïve (n = 2,500)**	**Colonoscopy-experienced (n = 500)**	**Odds ratio, with experience**
**It would be worth having a colonoscopy if it found a problem early**	82%	84%	1.147
	(77%–88%; France; Spain)	(82%–88%; France; Germany)	
**If my doctor recommended it, I would definitely have a colonoscopy**	72%	79%	1.460
	(63%–77%; France; Spain)	(72%–85%; France; Spain)	
**Even if I was worried or embarrassed, I would still have a colonoscopy**	67%	74%	1.452
	(56%–75%; France; Spain)	(63%–82%; France; Spain)	
**I would not tell anyone if I needed to have a colonoscopy**	25%	21%	0.806
	(17%–33%; Italy; UK)	(15%–33%; Germany; UK)	

#### Preferences for colonoscopy information

The doctor was the most common provider of information that colonoscopy-naïve respondents would seek guidance from if they wanted to know more (average: 80%, lowest: 70%, UK; highest: 86%, Spain). However, 41% of respondents would seek information from an internet search, 21% from family or friends, and 16% from a hospital website. Answers from fewer than 10% of respondents on where they would seek information were: a patient group website or helpline, and social media/forums.

Respondents indicated what methods of information dissemination would motivate them to attend a doctor-recommended colonoscopy (Table 3); the most common source being verbal advice directly from their doctor. Respondents also indicated their trust and mistrust of different sources of information about colonoscopy (Table 4); 87% of respondents would trust their doctor, and 55% would trust a hospital website. Thirty-nine percent of respondents would mistrust information from the internet.

**Table 3:** Respondents’ preferences for how to receive information, in order to improve motivation to attend colonoscopy (information in brackets signifies: Country with the lowest value; country with the highest value).

***Method of motivation***	***% of respondents choosing method***
*Verbal advice directly from my doctor (general practitioner)*	*73% (64%–82%; France; Spain)*
*Talking to someone who has had a colonoscopy*	*26% (12%–33%; Spain; UK)*
*A leaflet about what happens during a colonoscopy and the benefits of having the procedure*	*24% (16%–33%; Spain; UK)*
*Articles about the benefits of colonoscopy in newspapers*	*9% (7%–14%; Germany; UK)*
*Online videos about the benefits of colonoscopy*	*7% (4%–10%; Germany; UK)*
*Social media content posts shared by charities and the health service*	*5% (2%–7%; Germany; UK)*
*Celebrities speaking out about the benefits of colonoscopy*	*4% 3(%–7%; Italy; UK)*
*Adverts on TV*	*4% (2%–5%; Germany; UK)*
*None of the above*	*14% (9%–20%; Spain; France)*

**Table 4:** Trust in different sources to provide accurate information about colonoscopy (information in brackets signifies: Country with the lowest value; country with the highest value).

**Source**	**My doctor (general practitioner)**	**A patient group website**	**A patient group helpline**	**A hospital website**	**Internet**	**Family/ friends**
Would trust (% of respondents)	87%	43%	39%	55%	20%	45%
	(84%–90%; France; Italy)	(32%–54%; France; UK)	(24%–54%; France; UK)	(46%–71%; France; UK)	(15%–25%; France; UK)	(39%–49%; Spain; Germany)
Would not trust (% of respondents)	4%	20%	24%	14%	39%	20%
	(3%–5%; Spain; Germany)	(11%–32%; UK; France)	(12%–39%; UK; France)	(6%–21%; UK; France)	(30%–45%; UK; Spain)	(17%–23%; Germany; Spain)

#### Attitudes towards the colonoscopy procedure

Attitudes towards the colonoscopy procedure for colonoscopy-naïve and colonoscopy-experienced respondents, and odds ratios for attitudes of respondents with colonoscopy experience, are shown in Table 5. While a smaller proportion of the colonoscopy-experienced respondents had concerns about colonoscopy than colonoscopy-naïve respondents, some colonoscopy-experienced respondents would still be nervous, or embarrassed, about having a colonoscopy (49% and 43%, respectively). Thirty-six percent of colonoscopy-experienced patients had also been ‘worried about having a colonoscopy’ before their procedure, and 61% had been nervous at that time.

**Table 5:** Respondents’ attitudes towards the colonoscopy procedure (information in brackets signifies: The range of responses across the five countries surveyed; country with the lowest agreement with statement; country with the highest agreement with statement).

**Attitude to colonoscopy**	**Colonoscopy-naïve (n = 2,500)**	**Colonoscopy-experienced (n = 500)**	**Odds ratio, with experience**
**Would be nervous of having a colonoscopy**	74%	49%	0.334
	(69%–77%; UK; Italy)	(39%–62%; Germany; Spain)	
**Would be embarrassed to have a colonoscopy**	59%	43%	0.514
	(38%–78%; Germany; Spain)	(29%–66%; Germany; Spain)	
**Would be worried about a colonoscopy being painful**	59%	37%	0.412
	(55%–66%; Spain; UK)	(25%–48%; Germany; UK)	
**Would want to be unconscious**	56%	55%	0.956
	(41%–70%; UK; Spain)	(41%–69%; Germany; France)	

#### Attitudes towards the colonoscopy process: Perceived worst aspect

**Fig 2:**
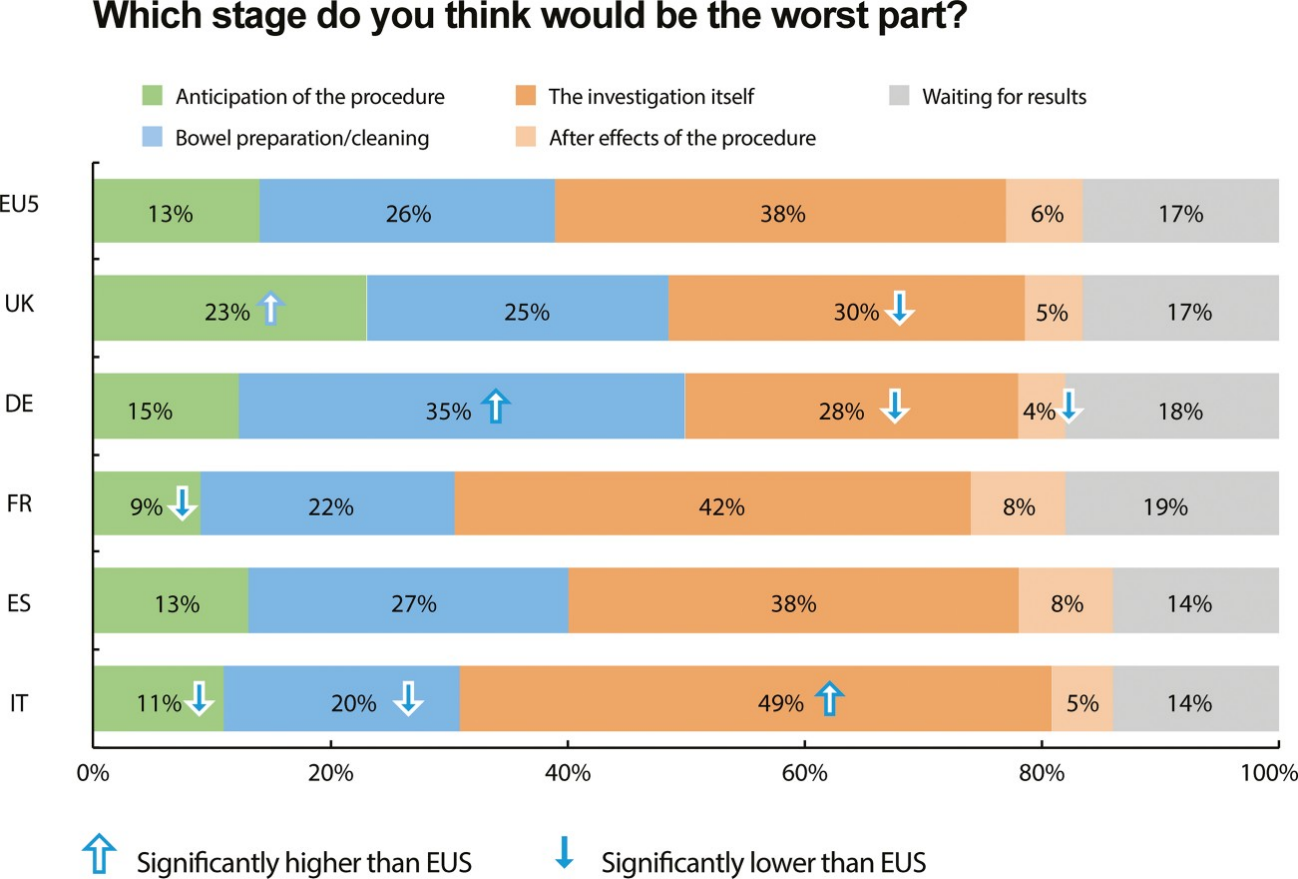
The stage of the colonoscopy procedure that is deemed the worst part by colonoscopy-naïve respondents (by country). Significance denotes p<0.05. EU5 = five European Union countries, UK = United Kingdom, DE = Germany, FR = France, ES = Spain, IT = Italy.

Thirty-eight percent of colonoscopy-naïve respondents believed that the colonoscopy procedure itself would be ‘the worst part’ of the process, while 26% believed that it would be the bowel preparation. However, there were significant variations in opinion between respondents from different countries (Fig 2). German participants in particular demonstrated a significantly greater aversion to bowel preparation compared with the EU5 average (accompanying a greater expectation of amount of fluid required, compared with the EU5 average; Fig 1). Perceptions of the worst part of the colonoscopy process are shown in Table 6, for both colonoscopy-naïve and colonoscopy-experienced respondents, alongside odds ratios for attitudes of respondents with colonoscopy experience. In every country, a greater proportion of colonoscopy-experienced respondents thought that ‘anticipation of the procedure’ and ‘bowel preparation / bowel cleaning’ were the worst parts of the process than colonoscopy-naïve respondents, while a lower proportion in every country thought that ‘the investigation itself’, ‘after effects of the procedure’ and ‘waiting for results’ were the worst parts of the process.

**Table 6:** Respondents’ perceptions of ‘the worst part’ of the colonoscopy process.

**Expectation/experience of ‘the worst part’ of the colonoscopy procedure**	**Colonoscopy-naïve (n = 2,500)**	**Colonoscopy-experienced (n = 500)**	**Odds ratio for this aspect vs others, with experience**
**Anticipation of the procedure**	14%	21%	1.643
**Bowel preparation/cleaning**	26%	47%	2.487
**The investigation itself**	38%	16%	0.322
**After effects of the procedure**	6%	4%	0.708
**Waiting for results**	17%	12%	0.677

#### Older patients (aged ≥41 years)

No substantial differences were observed between overall answers and answers from patients aged ≥41 years. For example, 83% agreed that ‘it would be worth having a colonoscopy if it found a problem early’ and 75% agreed that ‘if my doctor recommended it, I would definitely have a colonoscopy’ (vs 82% and 72% in all ages, respectively). Sixty-seven percent of respondents aged ≥41 years agreed that ‘even if I was worried or embarrassed, I would still have a colonoscopy’, and 21% ‘would not tell anyone’ if they needed to have a colonoscopy (vs 67% and 25% in all ages, respectively).

### (iv) Opinion of information provided before colonoscopy

For colonoscopy-experienced respondents, opinion of the information provided before colonoscopy (EU5 average, and by country) is shown in Table 7.

**Table 7:** Opinion of information provided before colonoscopy (agreement with statement, except where indicated).

**Region**	**EU5**	**UK**	**Germany**	**France**	**Spain**	**Italy**
**I had all the information I needed before the colonoscopy (agreement)**	**71%**	83%	80%	71%	62%	61%
**I had all the information I needed before the colonoscopy (disagreement)**	**10%**	1%	6%	6%	20%	15%
**I would have liked to discuss the colonoscopy more with a doctor or nurse**	**36%**	36%	27%	38%	42%	38%
**I would have liked more written information about what to expect during the colonoscopy**	**34%**	32%	22%	33%	41%	43%
**I would have liked more written information about bowel preparation**	**35%**	40%	21%	32%	38%	46%
**I would have liked to have spoken to someone who has had a colonoscopy (agreement)**	**33%**	37%	22%	29%	38%	37%
**I would have liked to have spoken to someone who has had a colonoscopy (disagreement)**	**40%**	31%	53%	50%	37%	29%

## Discussion

This was the first study that we know of to determine public attitudes towards the colonoscopy process, knowledge of the process, and preferences for learning about colonoscopy, and the first pan-European study to do so. Most cases of CRC are diagnosed after subjects experience symptoms and seek medical assessment, ultimately leading to a colonoscopy referral [17]. Nevertheless, in Europe CRC rates are increasing [2], and screening of asymptomatic elderly subjects is recommended in France, Germany, Italy, Spain, and the UK every two years, using (variously) fecal occult blood testing, fecal immunochemical testing, or flexible sigmoidoscopy [18]. While colonoscopy every 10 years is recommended in Germany for the elderly [18], the position of colonoscopy in the journey to CRC diagnosis is therefore typically as a referral procedure, rather than as a primary healthcare procedure. Nevertheless, the health literacy of the public towards CRC and attitudes to colonoscopy are still important, because misperceptions could delay attendance when it is required; delay in colonoscopy after an initial positive fecal occult blood test for example is associated with more advanced disease and higher CRC mortality [19].

Many findings in our suvey were expected, such as receptivity to colonoscopy after a doctor recommends it (72%), or the presence of educational gaps and embarrassment around colonoscopy. Disappointingly, 55% of our colonoscopy-naïve respondents did not know that colonoscopies are used to prevent bowel cancer (UK respondents in particular had very poor knowledge), while 43% of colonoscopy-experienced respondents would still be embarrassed of another colonoscopy. Previously reported educational gaps amongst outpatients have also included underestimation of CRC risk, overestimation of procedure risk, and underestimation of the success rate of treatment for early-stage CRC [15], while female patients in Italy [14] have expressed embarrassment at the idea of colonoscopy, and embarassment towards colonoscopy has been thoroughly characterized in the US [20].

In this study we did however discover misconceptions about the colonoscopy process, such as the concept of the ‘worst part’ of the colonoscopy process–colonoscopy-experienced respondents had 68% lower odds of believing that *the procedure itself* was the worst part (and fewer believed so in every country) than colonoscopy-naïve respondents. This misconception could be due to expectation of pain during the procedure, and/or underestimation of bowel preparation liquid required. First, pain control is a priority for all patients. The majority of both colonoscopy-naïve and colonoscopy-experienced respondents would want sedation for their next colonoscopy procedure, and a desire for pain control by both colonoscopy-naïve and colonoscopy-experienced outpatients (71% and 63% respectively, in this study) has been reported previously [15]. However, colonoscopy-naïve respondents tend to overestimate the likelihood of the procedure being painful (odds ratio, 0.50 with colonoscopy experience). It should be noted though that the pain experienced during a colonoscopy is dependent on sedation practices, and these vary between countries [21–23]: variations in practice include the safety methods used, practitioner type, and practioner knowledge of sedation guidelines [22]. In our survey, painful colonoscopy was experienced by 9% of German respondents, but 39% of Italian respondents; in Germany, over 75% of patients receive controlled sedation care, whereas in Italy, only 25–50% do [22].

Second, colonoscopy-naïve respondents may underestimate the discomfort of bowel preparation. The odds ratio of colonoscopy-experienced respondents believing that the ‘worst part’ of the colonoscopy process is the bowel preparation was 2.49 (and more believed so in every country) compared with colonoscopy-naïve respondents. Colonoscopy-naïve respondents tended to underestimate the bowel preparation volume needed: 67% thought that 1 litre of bowel preparation fluid or less needs to be drunk, whereas historically 2 litres or more of bowel preparation fluid has been required [24]. Respondents in Germany had higher expectations of the bowel preparation volume needed, and bowel preparation was accordingly the most common expectation for the ‘worst part’ of the colonoscopy process (35%, vs EU5 average of 26%; p<0.05).

We elucidated some differences in attitudes between countries. French respondents were often the least receptive to colonoscopy (Table 2) and least trustful of information about colonoscopy (Table 4). German respondents were often most receptive to colonoscopy (Table 2) and were least in need of speaking to someone about their colonoscopy before it had happened (Table 7). Italian colonoscopy-experienced respondents were most in want of more written information before colonoscopy, with the fewest feeling that they had received all the information they needed beforehand (Table 7). Spanish respondents were both the most receptive to (Table 2) and embarrassed about (Table 5) colonoscopy, but 20% of the colonoscopy-experienced respondents disagreed that they had received enough information beforehand, compared with the EU5 average of 10% disagreement (Table 7). Respondents in the UK were the most receptive and trustful of non-clinician education routes (Tables 3 and 4).

We can speculate that all subjects would benefit from knowing that pain during the colonoscopy procedure is overestimated (59% of colonoscopy-experienced respondents thought that the colonoscopy experience was better than expected). Further, although German subjects tend to know about fluid intake requirements, all patients would benefit from bowel preparation education, and perhaps Spanish and Italian patients in particular. Provision of details of the colonoscopy process is reported to improve compliance by two-to-three-fold [25–27]. In contrast, studies report that patients who find the instructions for bowel preparation ‘confusing’ are 3.5-fold more anxious about it than patients who find the instructions ‘somewhat clear’ [10], and underestimation of intake required deters compliance [16].

Given that 66% of colonoscopy-naïve patients rank bowel preparation as one of the top three reasons to not attend a colonoscopy, and a low volume bowel preparation as one of the top three methods to encourage attendance [15], practitioners may also wish to explore new bowel preparation techniques to help match low public expectations of fluid volume needed. A 1-litre bowel preparation, which has recently been approved in Europe [28–30], may for instance improve compliance. In any case, choices should be tailored for each patient based on bowel preparation volume, patients’ eating habits and daily activities [16].

The current study has limitations which may prevent the results representing the true attitudes of the wider public. There is selection bias, as respondents had replied to the survey for consumer reward and, although the quota sizes balanced statistical robustness and project manageability, the results have an intrinsic uncertainty due to the small number of respondents. Further, although the respondents in this survey were matched to represent the age, gender, administrative region, and occupational status demographics within their countries, there are other demographic variables influencing patient attidudes that were not matched, as these characteristics were not characterized in this survey; for example, educational background, income level, degree of familiarity with colorectal cancer and colonoscopy, and history of healthcare interventions [5,6,31]. There may also be systematic variations between countries, in healthcare education, and colonoscopy and bowel preparation methods for example, that may modulate attitudes and knowledge.

## Conclusions

This study demonstrated widespread low public understanding of the purpose of colonoscopy as both a diagnostic and a therapeutic tool against CRC throughout Europe, accompanied by poor provision of information, particularly in Italy and Spain. Misconceptions such as overestimating the discomfort of the colonoscopy procedure and underestimating the amount of bowel preparation fluid needed were found in all countries, though to a lesser degree in Germany. These misconceptions may supplement other known barriers to colonoscopy attendance, such as embarassment, and could be addressed by education, or use of lower-volume bowel preparations. However, other studies report a clear variability in preferences, knowledge and attitudes between patients, and while this survey was not statistically powered to identify precise differences between all countries and demographies, it is clear that a broad armamentarium of educational tools is prudent in order to appeal to different individuals, and to encourage attendance at colonoscopy referral [15].

## Supporting information

S1 Appendix Survey questions and answers. (DOCX)

S1 Data (ZIP)
