# Supplementary material for: A survey on colonoscopy shows poor understanding of its protective value and widespread misconceptions across Europe
Source: PLoS One. 2020 May 21;15(5):e0233490. doi: 10.1371/journal.pone.0233490 (PMC7241766; doi:10.1371/journal.pone.0233490)
Supplement: S1 Appendix — (DOCX) [file pone.0233490.s001.docx]

**Supplementary appendix – survey questions and answers**

**For all respondents**

1. What is your age?
2. What is your gender?
3. In what region do you live?
4. United Kingdom, North East
5. United Kingdom, Wales
6. United Kingdom, Scotland
7. United Kingdom, Northern Ireland
8. United Kingdom, North West
9. United Kingdom, Yorkshire and The Humber
10. United Kingdom, East Midlands
11. United Kingdom, West Midlands
12. United Kingdom, East of England
13. United Kingdom, London
14. United Kingdom, South East
15. United Kingdom, South West
16. Germany, Baden-Württemberg
17. Germany, Nordrhein-Westfalen
18. Germany, Rheinland-Pfalz
19. Germany, Saarland
20. Germany, Sachsen
21. Germany, Sachsen-Anhalt
22. Germany, Schleswig-Holstein
23. Germany, Thüringen
24. Germany, Bayern
25. Germany, Berlin
26. Germany, Brandenburg
27. Germany, Bremen
28. Germany, Hamburg
29. Germany, Hessen
30. Germany, Mecklenburg-Vorpommern
31. Germany, Niedersachsen
32. France, Île-de-France
33. France, Normandie
34. France, Bourgogne-Franche-Comté
35. France, Occitanie
36. France, Corse (Collectivité territoriale)
37. France, Auvergne-Rhône-Alpes
38. France, Nouvelle-Aquitaine
39. France, Hauts-de-France
40. France, Provence-Alpes-Côte d’Azur
41. France, Bretagne
42. France, Centre-Val de Loire
43. France, Pays de la Loire
44. France, Grand Est
45. Spain, Galicia
46. Spain, Castilla-la Mancha
47. Spain, Extremadura
48. Spain, Cataluña
49. Spain, Comunidad Valenciana
50. Spain, Illes Balears
51. Spain, Andalucía
52. Spain, Región de Murcia
53. Spain, Ciudad Autónoma de Melilla (ES)
54. Spain, Canarias (ES)
55. Spain, Principado de Asturias
56. Spain, Cantabria
57. Spain, País Vasco
58. Spain, Comunidad Foral de Navarra
59. Spain, La Rioja
60. Spain, Aragón
61. Spain, Comunidad de Madrid
62. Spain, Castilla y León
63. Italy, Piemonte
64. Italy, Calabria
65. Italy, Sicilia
66. Italy, Sardegna
67. Italy, Provincia Autonoma di Bolzano/Bozen
68. Italy, Provincia Autonoma di Trento
69. Italy, Veneto
70. Italy, Friuli-Venezia Giulia
71. Italy, Emilia-Romagna
72. Italy, Toscana
73. Italy, Umbria
74. Italy, Valle d'Aosta/Vallée d'Aoste
75. Italy, Marche
76. Italy, Lazio
77. Italy, Liguria
78. Italy, Lombardia
79. Italy, Abruzzo
80. Italy, Molise
81. Italy, Campania
82. Italy, Puglia
83. Italy, Basilicata
    1. Which of the following best describes your working status? United Kingdom
       1. Employee full-time
       2. Employee part-time (8–29 hours per week)
       3. Full-time education
       4. Full-time education (university)
       5. Retired
       6. Not able to work
       7. Unemployed and seeking work
       8. Not working for other reason
    2. Which of the following best describes your working status? Spain
       1. Working (full-time)
       2. Working (half-time)
       3. Retired/pensioner/disabled
       4. Unemployed, he/she worked before
       5. Unemployed, he/she is looking for his/her first job
       6. Student (does not work)
       7. Housewife (does not work)
    3. Which of the following best describes your working status? France
       1. Full-time profession
       2. Half-time profession
       3. Temporary unemployed or job seeking
       4. Pensioner, retiree
       5. Apprenticeship, pupil, student
       6. Occupational retraining
       7. Not working
    4. Which of the following best describes your working status? Italy
       1. Full-time working
       2. Part-time working
       3. Temporary unemployed or job seeking
       4. Pensioner, retiree
       5. Student
       6. Housewife
       7. Other (military, other conditions)
    5. Which of the following best describes your working status? Germany
       1. Full-time profession
       2. Half-time profession
       3. Temporary unemployed or job seeking
       4. Pensioner, retiree
       5. Apprenticeship, pupil, student
       6. Occupational retraining
       7. not working
84. Have you ever had a colonoscopy? If yes, was it more or less than 5 years ago?
    - 1. I have never had a colonoscopy
      2. I had a colonoscopy within the last 5 years
      3. I had a colonoscopy more than 5 years ago

**For colonoscopy-naïve respondents**

1. Which of the following statements best describes your level of knowledge of what colonoscopy is?
   - 1. I don´t know what colonoscopy is
     2. I know a little about what colonoscopy is
     3. I have a good idea about what colonoscopy is
     4. I have detailed knowledge of what colonoscopy is
2. Do you personally know anyone who has had a colonoscopy?
   - 1. Yes
     2. No
     3. Don´t know
3. Which of the following do you think colonoscopy is used for?
   - 1. Diagnosing diseases of the bowel
     2. Screening for bowel cancer
     3. Removal of an unusual growth in the bowel
     4. Preventing bowel cancer
     5. Ongoing monitoring of people who are known to have a bowel problem
     6. Radiology
     7. Abdominal surgery
     8. None of the above
4. How long do you think the colonoscopy investigation takes from the start to finish of the procedure?
   - 1. Less than 15 minutes
     2. Between 15 and 30 minutes
     3. Between 30 minutes and an hour
     4. More than an hour
     5. More than 2 hours
     6. More than 3 hours
     7. Don´t know
5. Please indicate how strongly you agree or disagree with the following statements:
   1. I would be nervous of having a colonoscopy
      1. 1. Strongly disagree (1)
      2. 2.- (2)
      3. 3.- (3)
      4. 4.- (4)
      5. 5. Strongly agree (5)
   2. I would be embarrassed to have a colonoscopy
      1. 1. Strongly disagree (1)
      2. 2.- (2)
      3. 3.- (3)
      4. 4.- (4)
      5. 5. Strongly agree (5)
   3. Having a colonoscopy is painful
      1. 1. Strongly disagree (1)
      2. 2.- (2)
      3. 3.- (3)
      4. 4.- (4)
      5. 5. Strongly agree (5)
   4. I would be worried about a colonoscopy being painful
      1. 1. Strongly disagree (1)
      2. 2.- (2)
      3. 3.- (3)
      4. 4.- (4)
      5. 5. Strongly agree (5)
   5. Even if I was worried or embarrassed, I would still have a colonoscopy
      1. 1. Strongly disagree (1)
      2. 2.- (2)
      3. 3.- (3)
      4. 4.- (4)
      5. 5. Strongly agree (5)
   6. I would not tell anyone if I needed to have a colonoscopy
      1. 1. Strongly disagree (1)
      2. 2.- (2)
      3. 3.- (3)
      4. 4.- (4)
      5. 5. Strongly agree (5)
   7. You need to be unconscious to have a colonoscopy
      1. 1. Strongly disagree (1)
      2. 2.- (2)
      3. 3.- (3)
      4. 4.- (4)
      5. 5. Strongly agree (5)
   8. I would want to be unconscious if I had a colonoscopy
      1. 1. Strongly disagree (1)
      2. 2.- (2)
      3. 3.- (3)
      4. 4.- (4)
      5. 5. Strongly agree (5)
   9. If my doctor recommended it, I would definitely have a colonoscopy
      1. 1. Strongly disagree (1)
      2. 2.- (2)
      3. 3.- (3)
      4. 4.- (4)
      5. 5. Strongly agree (5)
   10. It would be worth having a colonoscopy if it found a problem early
       1. 1. Strongly disagree (1)
       2. 2.- (2)
       3. 3.- (3)
       4. 4.- (4)
       5. 5. Strongly agree (5)
6. How much of the bowel preparation liquid do you think a patient needs to drink prior to the procedure?
   - 1. Less than 500ml (less than a pint)
     2. Less than 1 litre (less than 2 pints)
     3. 1 litre (approximately 2 pints)
     4. 2 litres (approximately 4 pints)
     5. More than 2 litres (more than 4 pints)
7. From what you know about colonoscopy, which stage of the colonoscopy procedure do you think would be the worst part?
   - 1. Anticipation of the procedure
     2. Bowel preparation/bowel cleaning
     3. The investigation itself
     4. After-effects of the procedure
     5. Waiting for results
8. Which of the following would make you more likely to attend a colonoscopy appointment if your doctor thought it was appropriate?
   - 1. Verbal advice directly from my doctor (general practitioner)
     2. Talking to someone who has had a colonoscopy
     3. Articles about the benefits of colonoscopy in newspapers
     4. Celebrities speaking out about the benefits of colonoscopy
     5. A leaflet about what happens during a colonoscopy and the benefits of having the procedure
     6. Adverts on TV
     7. Online videos about the benefits of colonoscopy
     8. Social media content posts shared by charities and the health service
     9. None of the above
9. If you wanted more information about colonoscopy, where would you go to look for it?
   - 1. My doctor (general practitioner)
     2. General internet search
     3. A hospital website
     4. A patient group website
     5. A patient group helpline
     6. Family/friends
     7. Social media or forums
     8. Other
10. To what extent would you trust each of the following to give you accurate information about colonoscopy?
    1. My doctor (general practitioner)
       1. 1. Would not trust at all (1)
       2. 2.- (2)
       3. 3.- (3)
       4. 4.- (4)
       5. 5. Completely trust (5)
    2. The internet
       1. 1. Would not trust at all (1)
       2. 2.- (2)
       3. 3.- (3)
       4. 4.- (4)
       5. 5. Completely trust (5)
    3. A hospital website
       1. 1. Would not trust at all (1)
       2. 2.- (2)
       3. 3.- (3)
       4. 4.- (4)
       5. 5. Completely trust (5)
    4. A patient group website
       1. 1. Would not trust at all (1)
       2. 2.- (2)
       3. 3.- (3)
       4. 4.- (4)
       5. 5. Completely trust (5)
    5. A patient group helpline
       1. 1. Would not trust at all (1)
       2. 2.- (2)
       3. 3.- (3)
       4. 4.- (4)
       5. 5. Completely trust (5)
    6. Family/friends
       1. 1. Would not trust at all (1)
       2. 2.- (2)
       3. 3.- (3)
       4. 4.- (4)
       5. 5. Completely trust (5)

**For colonoscopy-experienced respondents**

1. How long is it since you had your colonoscopy? If you had more than 1 colonoscopy in the past 5 years, please answer for the most recent one.
   - 1. Less than a year
     2. Between 1 and 2 years
     3. Between 2 and 3 years
     4. Between 3 and 4 years
     5. Between 4 and 5 years
2. What prompted you to have a colonoscopy?
   - 1. I was called in for routine screening
     2. I was referred for a colonoscopy because I was having symptoms
     3. I am required to have a regular colonoscopy due to an ongoing health condition
     4. Other
     5. Don´t know
3. Which of the following do you think colonoscopy is used for?
   - 1. Diagnosing diseases of the bowel
     2. Screening for bowel cancer
     3. Removal of an unusual growth in the bowel
     4. Preventing bowel cancer
     5. Ongoing monitoring of people who are known to have a bowel problem
     6. None of the above
4. Before your colonoscopy, did you do any of the following?
   1. Talk to a doctor about what the procedure would involve
      1. Yes
      2. No
      3. Don’t know
   2. Talk to a nurse about what the procedure would involve
      1. Yes
      2. No
      3. Don’t know
   3. Talk to someone who had already had a colonoscopy
      1. Yes
      2. No
      3. Don’t know
   4. Read a leaflet about it
      1. Yes
      2. No
      3. Don’t know
   5. Look online for information
      1. Yes
      2. No
      3. Don’t know
5. Thinking back, please indicate how strongly you agree or disagree with the following statements?
   1. I was not worried about having a colonoscopy
      1. 1. Strongly disagree (1)
      2. 2.- (2)
      3. 3.- (3)
      4. 4.- (4)
      5. 5. Strongly agree (5)
   2. I was nervous before my colonoscopy
      1. 1. Strongly disagree (1)
      2. 2.- (2)
      3. 3.- (3)
      4. 4.- (4)
      5. 5. Strongly agree (5)
   3. I was embarrassed to have a colonoscopy
      1. 1. Strongly disagree (1)
      2. 2.- (2)
      3. 3.- (3)
      4. 4.- (4)
      5. 5. Strongly agree (5)
   4. I was afraid the colonoscopy would hurt
      1. 1. Strongly disagree (1)
      2. 2.- (2)
      3. 3.- (3)
      4. 4.- (4)
      5. 5. Strongly agree (5)
6. When you had your colonoscopy, did you have:
   - 1. Full sedation so you were asleep
     2. Conscious sedation so you had sedation but were not fully asleep
     3. Local anaesthetic so you were conscious
     4. No sedation or local anaesthetic
     5. Don´t know/can´t remember
7. Thinking back to your colonoscopy, which stage of the procedure do you think was the worst part?
   - 1. Anticipation of the procedure
     2. Bowel preparation/bowel cleaning
     3. The investigation itself
     4. After-effects of the procedure
     5. Waiting for results
8. Was your experience of having a colonoscopy:
   - 1. Much better than I expected
     2. A bit better than I expected
     3. As I expected
     4. Worse than I expected
     5. Much worse than I expected
9. Thinking back to the information you got about colonoscopy, please indicate how strongly you agree or disagree with the following statements:
   1. I had all the information I needed before the colonoscopy
      1. 1. Strongly disagree (1)
      2. 2.- (2)
      3. 3.- (3)
      4. 4.- (4)
      5. 5. Strongly agree (5)
   2. I would have liked more written information about what to expect during the colonoscopy
      1. 1. Strongly disagree (1)
      2. 2.- (2)
      3. 3.- (3)
      4. 4.- (4)
      5. 5. Strongly agree (5)
   3. I would have liked more written information about bowel preparation
      1. 1. Strongly disagree (1)
      2. 2.- (2)
      3. 3.- (3)
      4. 4.- (4)
      5. 5. Strongly agree (5)
   4. I would have liked to discuss the colonoscopy more with a doctor or nurse
      1. 1. Strongly disagree (1)
      2. 2.- (2)
      3. 3.- (3)
      4. 4.- (4)
      5. 5. Strongly agree (5)
   5. I would have liked to have spoken to someone who has had a colonoscopy
      1. 1. Strongly disagree (1)
      2. 2.- (2)
      3. 3.- (3)
      4. 4.- (4)
      5. 5. Strongly agree (5)
10. Please indicate how strongly you agree or disagree with the following statements based on your experience of previously having had a colonoscopy:
    1. I would be nervous of having a colonoscopy
       1. 1. Strongly disagree (1)
       2. 2.- (2)
       3. 3.- (3)
       4. 4.- (4)
       5. 5. Strongly agree (5)
    2. I would be embarrassed to have a colonoscopy
       1. 1. Strongly disagree (1)
       2. 2.- (2)
       3. 3.- (3)
       4. 4.- (4)
       5. 5. Strongly agree (5)
    3. Having a colonoscopy is painful
       1. 1. Strongly disagree (1)
       2. 2.- (2)
       3. 3.- (3)
       4. 4.- (4)
       5. 5. Strongly agree (5)
    4. I would be worried about a colonoscopy being painful
       1. 1. Strongly disagree (1)
       2. 2.- (2)
       3. 3.- (3)
       4. 4.- (4)
       5. 5. Strongly agree (5)
    5. Even if I was worried or embarrassed, I would still have a colonoscopy
       1. 1. Strongly disagree (1)
       2. 2.- (2)
       3. 3.- (3)
       4. 4.- (4)
       5. 5. Strongly agree (5)
    6. I would not tell anyone if I needed to have a colonoscopy
       1. 1. Strongly disagree (1)
       2. 2.- (2)
       3. 3.- (3)
       4. 4.- (4)
       5. 5. Strongly agree (5)
    7. You need to be unconscious to have a colonoscopy
       1. 1. Strongly disagree (1)
       2. 2.- (2)
       3. 3.- (3)
       4. 4.- (4)
       5. 5. Strongly agree (5)
    8. You need to be conscious to have a colonoscopy
       1. 1. Strongly disagree (1)
       2. 2.- (2)
       3. 3.- (3)
       4. 4.- (4)
       5. 5. Strongly agree (5)
    9. I would want to be unconscious if I had a colonoscopy
       1. 1. Strongly disagree (1)
       2. 2.- (2)
       3. 3.- (3)
       4. 4.- (4)
       5. 5. Strongly agree (5)
    10. If my doctor recommended it, I would definitely have a colonoscopy
        1. 1. Strongly disagree (1)
        2. 2.- (2)
        3. 3.- (3)
        4. 4.- (4)
        5. 5. Strongly agree (5)
    11. It would be worth having a colonoscopy if it found a problem early
        1. 1. Strongly disagree (1)
        2. 2.- (2)
        3. 3.- (3)
        4. 4.- (4)
        5. 5. Strongly agree (5)
    12. I would encourage my family or friends to have a colonoscopy if their doctor recommended it
        1. 1. Strongly disagree (1)
        2. 2.- (2)
        3. 3.- (3)
        4. 4.- (4)
        5. 5. Strongly agree (5)
